# Exploring the Molecular Distributions in Dilute Polymer Solutions Using a Multi-Scale Numerical Solver

**DOI:** 10.3390/polym10040387

**Published:** 2018-04-01

**Authors:** Yi Liu, Canqun Yang, Cheng-Kun Wu, Xiang Zhang, Xin Zhang, Xiao-Wei Guo

**Affiliations:** 1 College of Computer, National University of Defense Technology, No. 109 Deya Street, Changsha 410073, China; liuyi16@nudt.edu.cn (Y.L.); canqun@nudt.edu.cn (C.Y.); chengkun_wu@nudt.edu.cn (C.-K.W.); zhangxiang08@nudt.edu.cn (X.Z.); 2 PLA Daily, No. 34 Fuwai Street, Beijing 100832, China; jfjbgfjg@163.com

**Keywords:** dilute polymer solutions, multi-scale simulation, molecular distributions, OpenFOAM

## Abstract

Simulating the rheological behaviors of polymer solutions is intrinsically a multi-scale problem. To study the macroscopic and microscopic characteristics in the fluid flow of dilute polymer solutions, we designed a multi-scale solver, which couples the Brownian Configuration Fields with the macroscopic hydrodynamic governing equations. Numerical simulation results using the multi-scale solver exhibited good accordance with the macroscopic only approach. Through a scalar field *D* we also quantitatively studied the flow behaviours in 2D planar channels, and analyzed the correlation between the molecular distribution and the macroscopic fluid flow in polymer solutions. Our results verified the correctness of the solver, which could provide valuable guidance for multi-scale simulations of complex fluids based on OpenFOAM.

## 1. Introduction

Polymer solutions are frequently used in the production of fibers, films, glues, lacquers, paints, and other items made of polymer materials. It is of great scientific significance and application values to make an in-depth study of the dynamics and the rheological characteristics of the polymer solutions through numerical simulation.

Simulating the rheological behaviors of polymer solutions is intrinsically a multi-scale problem. Multi-scale simulation is a rapidly growing multidisciplinary field, as stated in the review article provided in [1].

Numerical approaches for simulating polymer solutions fall into three categories: the macroscopic, the microscopic and the micro-macro multi-scale methods. In a macroscopic only simulation, the constitutive equation (CE) that relates the viscoelastic stress to the deformation history can be derived from the continuum mechanics. One then solves the constitutive model together with the conservation laws of mass and momentum to predict velocity and stress fields in complex flows. An extensive description of constitutive models is given by Owens and Phillips [2]. The Oldroyd-B model originated from a two-bead dumbbell model with a linear Hookean spring force is employed in our simulations to compute the stress tensor. Dumbbell models ignore the interactions among polymer molecules and they are usually employed to simulate the dilute polymer solutions. Multi-bead chains have been studied as well [3,4]. There are also other constitutive models such as the FENE-P (the closure approximation for finitely extensible nonlinear elastic dumbbell model proposed by Peterlin) model [5], the FENE-L (the closure approximation for finitely extensible nonlinear elastic dumbbell model proposed by Lielens) model and the FENE-LS (the simplification of closure approximation for finitely extensible nonlinear elastic dumbbell model proposed by Lielens) model [6], which are simplifications of a finitely extensible nonlinear elastic (FENE) spring. A macroscopic computational model is fast and simple. Nevertheless, it fails to reproduce many complex phenomena observed in experiments since the macroscopic model ignores microscopic details of molecular dynamics [7].

In addition, derivation of a constitutive equation from the kinetic theory [8,9] usually involved closure approximations. In general, constitutive equations cannot be derived exactly from the kinetic theory due to the so-called closure problem. Moreover, closure approximations have manifest effects on the behavior of the model, and sometimes they can even lead to unphysical predictions [10]. Although the FENE-L and FENE-LS approximations in [6] give accurate simulations while avoiding such unphysical phenomena, more reliable schemes rather than FENE-LS have been proposed [11,12,13,14], even for strong flows [15].

As a microscopic approach, the atomistic modelling could provide the most detailed description of the fluids dynamics. And the microscopic approaches were generally used to study the behaviour of polymers in the vicinity of solid walls and geometrical singularities such as a reentrant corner. This method is also employed to study the phenomena of wall slip and rupture [16,17,18]. However, such simulations require a lot more computing resources compared to their macroscopic counterparts. Thus the microscopic approach is currently limited to flow geometries of molecular dimensions.

The micro-macro techniques couple the coarse-grained molecular scale kinetic theory into the macroscopic continuum mechanics. In a micro-macro simulation, representing polymer molecules by a system of beads connected with massless springs leads to the Fokker-Planck equation, which governs the evolution of the distribution function [19]. Then, the Kramers’ formula relates the viscoelastic stress to the distribution function [20,21]. The Fokker-Planck equation, Kramers’ formula and conservation equation, supplemented with suitable initial and boundary conditions, constitute the generic multi-scale formulation of polymer solutions. Although these methods require a lot more computing resources than conventional continuum computations, they directly employ kinetic theory models in flow simulations, thus avoiding potentially inaccurate closure approximations.

Based on the theory of stochastic calculus, stochastic approaches replace the Fokker-Planck equation with a mathematically equivalent stochastic differential equation, in which a Wiener process models the Brownian forces acting on the polymer. Although the numerical solution to stochastic difference equations usually introduces noises, it is more applicable in the case of higher Weissenberg or Deborah number flows than comparable methods [22]. The first implementation of the stochastic approach is the CONNFFESSIT (Calculation of Non-Newtonian Flow: Finite Elements and Stochastic Simulation Techniques) method introduced by Öttinger and Laso [23]. At the beginning of a simulation, a large number of sample particles are distributed uniformly over the entire flow domain. These particles, each representing a polymeric configuration, are used for numerical approximation to the stochastic process. And they are convected along flow trajectories as the simulation proceeds. The stress tensor can be obtained through a Monte-Carlo integration. However, wild spatial oscillations are displayed in the stress fields due to a non-uniform particle density and uncorrelated Brownian forces acting on individual sample particles [24,25]. Prieto and Ellero also coupled the stochastic simulations of polymer kinetic models to macroscopic flow solvers [26,27].

The Brownian configuration field (BCF) method by Hulsen et al. [28] made a breakthrough via the use of correlated local ensembles. The BCF method initially defines the same local ensemble of sample particles within each material element. Thus, it ensures a homogeneous and dilute polymeric density in the physical space. Moreover, the correlated local Brownian forces lead to a uniform stress tensor field in the flow space. By reducing the global accuracy of the stress tensor field, the BCF method produces spatial smoothness in the stress and velocity fields. As a result, the stability of the numerical solution increases considerably [29,30].

A large number of micro-macro simulations have been conducted to study the flow characteristics of polymer solutions [1], but no systematic analysis was provided on the correlation between the molecular distributions and the macroscopic fluid flow. In this paper, we aim to demonstrate the possibility of exploring the microscopic characteristics using micro-macro simulations.

In the remaining of this paper, we first present the governing equations on the macro- and micro-scale in Section 2. Then, Section 3 discusses the spatial and temporal numerical discretization schemes of the Navier-Stokes, Oldroyd-B and stochastic differential equations. In Section 4, we give the implementation of a multi-scale numerical solver, BCFsolver. The mesh geometry and initial conditions used in the simulations, along with the numerical results, are presented in Section 5. At last, we validate our findings and discuss potential extensions.

## 2. Governing Equations

The flow of incompressible and isothermal dilute polymer solutions could be governed by the macroscopic and the microscopic description, respectively. In this section the momentum balance equation, the continuity equation and the stress tensor equation are first presented on the macro-scale, then a Fokker-Planck and a stochastic differential equation are employed respectively to replace the macroscopic stress tensor equation on the micro-scale.

### 2.1. Macroscopic Equations

We consider the isothermal and incompressible fluid of polymer solutions with density ρ. From the macroscopic perspective, the motion of a polymer solution fluid can be governed by the momentum balance and the continuity equation:

(1) $$\rho \left(\frac{\partial u}{\partial t}+u\cdot \nabla u\right)=-\nabla p+\eta_{s}\Delta u+\nabla \cdot \tau_{p}$$

(2) ∇·u=0

where $\eta_{s}$ is the solvent viscosity and $\tau_{p}$ is the time-dependent viscoelastic stress contributed from the polymer dynamics. The u and *p* are the velocity of the fluid and the pressure field, respectively.

The polymer contribution to the stress can be calculated through a constitutive equation. On the macro-scale, the Oldroyd-B model is considered. The Oldroyd-B constitutive equation [31] can be expressed as

(3) $$\tau_{p}+\lambda \overset{\nabla }{\tau_{p}}=2\eta_{p}D$$

where the symmetric deformation tensor $D=\frac{1}{2}\left(\nabla u+{\left(\nabla u\right)}^{T}\right)$, λ is the relaxation time of the dumbbell system and $\eta_{p}$ is the polymeric viscosity. The upper convected derivative of the polymer stress tensor $\overset{\nabla }{\tau_{p}}$ [31], is expressed as

(4) $$\overset{\nabla }{\tau_{p}}\equiv \frac{\partial \tau_{p}}{\partial t}+\left(u\cdot \nabla \right)\tau_{p}-{\left(\nabla u\right)}^{T}\cdot \tau_{p}-\tau_{p}\cdot \nabla u$$

The set of coupled Equations (1)–(3), supplemented with suitable initial and boundary conditions is the so-called macroscopic formulation of viscoelastic flows.

By scaling the equations with the characteristic units $L_{c}$ (characteristic length in macroscopic flow), $U_{c}$ (characteristic fluid velocity), $\rho_{c}$ (fluid density, scaling pressure term with $\left.1/(\rho U_{c}^{2}\right.)$) and normalizing the polymeric stress tensor with $\left.L_{c}/\left(U_{c}\left(\eta_{s}+\eta_{p}\right)\right)\right.$, Equations (1)–(3) can be rewritten in a dimensionless form as:

(5) $$\frac{\partial u}{\partial t}+u\cdot \nabla u=-\nabla p+\frac{1}{Re}\beta \Delta u+\frac{1}{Re}\nabla \cdot \tau_{p}$$

(6) ∇·u=0

(7) $$\tau_{p}+De\overset{\nabla }{\tau_{p}}=2\left(1-\beta \right)D$$

in which the dimensionless parameters De (Deborah number), Re (Reynolds number) and β (viscosity ratio) are defined as

(8) $$Re=\frac{\rho_{c}U_{c}L_{c}}{\eta_{s}+\eta_{p}},\;De=\frac{\lambda U_{c}}{L_{c}},\;\beta =\frac{\eta_{s}}{\eta_{s}+\eta_{p}}.$$

For simplicity, the same notation for the knowns are used in the dimensionless formulation as in (1)–(3).

### 2.2. Microscopic Equations

From a microscopic viewpoint, the Oldroyd-B equation originates from a Hookean dumbbell model. In this model, the molecules in polymer solution are represented by massive elastic dumbbells. All of them consist of two Brownian beads connected with a linear spring. The spring denotes inter-molecular forces between the two beads. The length and orientation of the spring describe a dumbbell’s configuration indicated by a vector $\tilde{q}$, which has dimension of length. The spring force reads as $\tilde{F}\left(\tilde{q}\right)=C\tilde{q}$ (*C* is the spring constant). In the remainder of this paper, a dumbbell’s configuration vector $\tilde{q}$ and the spring force $\tilde{F}\left(\tilde{q}\right)$ are scaled as dimensionless quantity q and F(q), respectively. Refer to [28] for more detailed information about how the models are nondimensionalized.

#### 2.2.1. Fokker-Planck Approach

The probability of finding a dumbbell with a configuration q at (r,t) is indicated by ψ(r,q,t). ψ is a probability density function (pdf) and fulfills ψ(r,q,t)≥0, ∫ψ(r,q,t)dq=1 in the geometrical domain and configuration space at any time of the simulation. By applying Newton’s second law to the forces acting on a dumbbell system, we can get the dimensionless Fokker-Planck equation [31].

(9) $$\frac{\partial \psi }{\partial t}+\nabla_{r}\cdot \left(u\psi \right)+\nabla_{q}\cdot \left({\left(\nabla_{r}u\right)}^{T}q\psi -\frac{1}{2De}F\left(q\right)\psi \right)=\frac{1}{2De}\Delta_{q}\psi$$

The Equation (9) describes the evolution of ψ under the dumbbell’s spring force F(q). In Equation (9) we dropped the diffusion term $\Delta_{r}\psi$ in physical space because the diffusion coefficient scales quadratically in the micro-macro length scale ratio $\left.L_{c}/l_{c}\right.$ and is usually only of the order $10^{-8}$ [32].

In this paper, we employed two different spring forces [31] to characterize elastic forces:

(10) F(q)=q,(Hooke)

(11) $$F\left(q\right)=\frac{q}{1-{∥q∥}^{2}/b},\;{∥q∥}^{2}\leq b\;\left(\mathrm{FENE}\right)$$

where $b=\left.∥q_{\max}{∥}^{2}/l_{c}\right.$ is dimensionless unit for the dumbbell’s maximum extension $q_{\max}$ compared to a characteristic micro length $l_{c}$. Note that for the FENE spring force in Equation (11), the dumbbell’s extension is restricted.

The pdf ψ in Equation (9) represents the polymeric configuration of the micro-system. Now Kramers’ equation [31] reads as:

(12) $$\tau_{p}=\frac{\alpha_{b,d}\left(1-\beta \right)}{De}\left(\langle q⊗F\left(q\right)\rangle -I\right)$$

where 〈·〉=∫·ψ(r,q,t)dq denotes the expectation in configuration space and I is a unity matrix [28]. The prefactor $\alpha_{b,d}$ specifies a spring dependent constant and is defined as

(13) $$\alpha_{b,d}\equiv \left\{\begin{array}{c} 1,\;\mathrm{for}\;\mathrm{Hookean}\;\mathrm{dumbbells}\;(b\to \infty ),\\ \frac{b+d+2}{b},\;\mathrm{for}\;\mathrm{FENE}\;\mathrm{dumbbells}\;(d\;\mathrm{is}\;\mathrm{the}\;\mathrm{dimension}\;\mathrm{of}\;\mathrm{configuration}\;\mathrm{space}). \end{array}\right.$$

The conservation of angular momentum leads to a symmetric $\tau_{p}$.

#### 2.2.2. Stochastic Brownian Configuration Field Approach

The BCF method replaces the Fokker-Planck equation with a corresponding stochastic differential equation [31].

(14) $$dQ_{t}\left(r\right)=\left(-u\cdot \nabla Q_{t}\left(r\right)+{\left(\nabla u\right)}^{T}\cdot Q_{t}\left(r\right)-\frac{1}{2De}F\left(Q_{t}\left(r\right)\right)\right)dt+\sqrt{\frac{1}{De}}dW_{t}$$

where $Q_{t}$ is a stochastic process and represents the configuration vector q. The 3-dimensional Gaussian Wiener process $W_{t}$ used to model Brownian forces is described by its first and second moments $\langle W_{t}\rangle =0$ and $\langle W_{t}W_{t}^{\prime }\rangle =min\left(t,t^{\prime }\right)I$. To approximate the actual probability density function, we solve Equation (14) for a number of stochastic realizations, namely Brownian configuration fields, represented by $Q_{t}^{\left(i\right)},i=1,2,\cdots ,N_{\mathrm{BCF}}$. Using a Monte Carlo integration, the first moment $\langle Q_{t}⊗F\left(Q_{t}\right)\rangle$ in Kramers’ Equation (12) can be approximately calculated by

(15) $$\langle Q_{t}⊗F\left(Q_{t}\right)\rangle \approx \frac{1}{N_{\mathrm{BCF}}}\left(\sum_{i=1}^{N_{\mathrm{BCF}}}Q_{t}^{\left(i\right)}⊗F\left(Q_{t}^{\left(i\right)}\right)\right)$$

As a result, the polymer contribution to the extra-stress $\tau_{p}$ can be given by

(16) $$\tau_{p}\approx \frac{\alpha_{b,d}\left(1-\beta \right)}{De}\left(\frac{1}{N_{\mathrm{BCF}}}\left(\sum_{i=1}^{N_{\mathrm{BCF}}}Q_{t}^{\left(i\right)}⊗F\left(Q_{t}^{\left(i\right)}\right)\right)-I\right)$$

From Equation (16), we can conclude that the accuracy of $\tau_{p}$ and the computing time critically depend on the choice of $N_{\mathrm{BCF}}$.

## 3. Numerical Methods

The momentum, continuity and constitutive equations are discretized through the finite volume method, which computes each term of the governing equations via volume integral over a control volume, which assumes a local conservation of physical laws. The 2nd-order Gauss MINMOD and Gauss linear scheme are employed to discretize the spatial terms. Temporal terms are discretised through a simple Euler scheme. The resulting equations would finally reduce to linear systems. As a result, we can get the solutions of these equations at each time step through iterative solvers predefined in OpenFOAM. The conjugate (PCG) and biconjugate gradient (PBiCG) methods are typical linear solvers in the toolbox. More details can be derived from the OpenFOAM Manual [33].

The discrete elastic-viscous split stress (DEVSS) numerical strategy of Guénette and Fortin [34] was employed to solve the governing equations presented in Section 2. And the numerical method modifies the PISO (pressure-implicit split-operator) algorithm by explicitly introducing polymer stress unknowns into the momentum equation as source terms. The momentum equation is rewritten as

(17) $$\frac{\partial u}{\partial t}+u\cdot \nabla u-\frac{\eta_{s}}{\rho }\Delta u-\frac{\nabla \cdot \tau_{p}}{\rho }=-\frac{\nabla p}{\rho }$$

Discretized through finite volume method, this equation can be written as a linear system:

(18) $$Au^{\left(n+1\right)}=H^{n}-\nabla {\left[p\right]}^{n}$$

where the polymer stress contribution is included as a source term in the symbol $H^{n}$. The convective and viscous terms are implicitly discretized into the matrix A of the linear system. The square brackets [·] indicates numerical approximation of the corresponding variable; *n* and n+1 signify the past and present times of the variable, respectively. Multiplying Equation (18) by $A^{-1}$ yields

(19) $$u^{\left(n+1\right)}=U^{\left(n+1\right)}-A^{-1}\nabla {\left[p\right]}^{n}$$

where $U^{\left(n+1\right)}=A^{-1}H^{n}$. A Poisson equation is derived as Equation (20) for solving the pressure in the pressure-correction step by taking the divergence of Equation (19) and applying the continuity equation (∇·u=0).

(20) $$\nabla \cdot U^{\left(n+1\right)}=\nabla \cdot {\left(A^{-1}\nabla \left[p\right]\right)}^{n+1}$$

Equations (19) and (20) are the key steps for the PISO algorithm. In the BCFsolver, the consitutive equation part is replaced with a microscopic BCF method to solve the viscoelastic stress tensor. A semi-implicit Eular method is employed to solve the equations of the Brownian configuration fields. The Equation (14) can be rewritten as

(21) $$\begin{array}{cc} \left(1+\frac{\Delta t^{n}}{2De(1-∥Q_{n+1}^{\left(i\right)}\left(r\right){∥}^{2}/b)}\right)Q_{n+1}^{\left(i\right)}\left(r\right) & =Q_{n}^{\left(i\right)}\left(r\right)+\left(-u^{n}\cdot \nabla Q_{n}^{\left(i\right)}\left(r\right)+{\left(\nabla u^{n}\right)}^{T}\cdot Q_{n}^{\left(i\right)}\left(r\right)\right)\Delta t^{n}\\ & +\sqrt{\frac{\Delta t^{n}}{De}}N{\left(0,1\right)}^{\left(i\right)} \end{array}$$

Equation (21) is used to get the configuration field $Q_{i}\left(r,t\right)$ at time $t^{n+1}$ for the FENE spring force. In case of the Hookean spring force, replace the $2De(1-∥Q_{n+1}^{\left(i\right)}\left(r\right){∥}^{2}/b)$ with 2De. N(0,1) denotes a triple of independent Gaussian random variables with zero mean and variance one.

To compute the polymer stress tensor, Equation (16) can be rewritten using semi-implicit Eular method as

(22) $$\tau_{p}^{n+1}\left(r\right)=\frac{\alpha_{b,d}\left(1-\beta \right)}{De}\left(\frac{1}{N_{\mathrm{BCF}}}\left(\sum_{i}Q_{n+1}^{\left(i\right)}\left(r\right)⊗F\left(Q_{n+1}^{\left(i\right)}\left(r\right)\right)\right)-I\right)$$

Coupling the macroscopic and microscopic parts, the iterative algorithm for solving the multi-scale model is summarised as Algorithm 1.

**Algorithm 1:** The iterative algorithm to solve the multi-scale model.
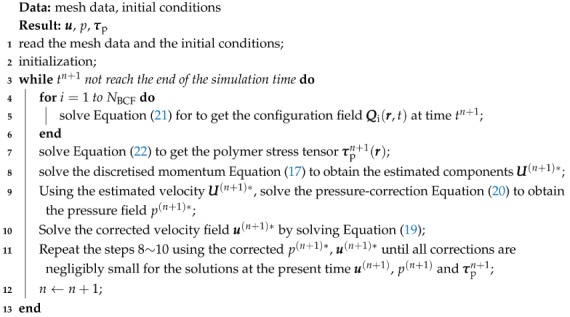


## 4. BCFsolver Implementation

To solve the micro-macro governing equations, we implement a multi-scale solver named BCFsolver according to the numerical Algorithm 1 mentioned above. In this section, we specify the implementation process of the BCFsolver.

### 4.1. Overall Structure

OpenFOAM is a free, open source platform that not only provides us with a rich set of application solvers but also allows users to write custom solvers for specific applications based on their original solvers. It is often used to study the fluid dynamics of polymer solutions [35,36]. OpenFOAM is written in C++ and provides user interfaces that can describe partial differential equations in a natural language-like way, which makes it easy to extend the theoretical models of existing solvers.

**Figure 1:**
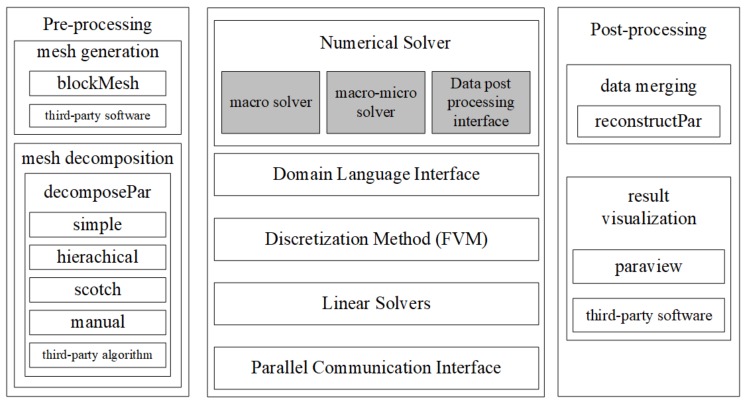
Numerical solver system organization.

The system organization of the BCFsolver based on the OpenFOAM is described in Figure 1. The solver module designed by us based on the OpenFOAM application framework is mainly the part deepened in gray.

According to the solution process, the entire numerical solution process includes three parts: pre-processing, numerical solution and post-processing. The pre-processing part performs mesh generation and mesh decomposition. Mesh generation breaks down a continuous geometric space into tiny grids, and the “blockMesh” tool generates corresponding grids according to the input configuration files. For complex geometries, OpenFOAM can import a mesh generated by third-party software tools. Mesh decomposition will decompose the grid into multiple parts for parallel computing.

The post-processing part mainly includes the data merging and the visualization of the results. After parallel computation, the results are distributed in the corresponding folders of each processor. Data merging consolidates the corresponding calculation results to analyse using the “reconstructPar” tool. OpenFOAM integrates an open source visualization software, “paraView” (5.0.1, Kitware lnc., Clifton Park, NY, USA), which enables basic presentation and analysis of results. At the same time, OpenFOAM can also output a variety of different data formats for third-party software to visualize.

The numerical solution includes four layers: the domain language interface, the discretization method, the linear solvers and the parallel communication interfaces. The parallel communication interfaces are developed based on the MPI library and provides communication facilities in the form of APIs (Application Programming Interface). A variety of parallel solvers are used to solve linear systems, and the iterative solution of a linear system is achieved by calling the underlying parallel communication interface. By using the FVM (Finite Volume Method) method, the differential equations are discretized into the form of a linear system. Domain language interfaces are the key to writing complex fluid solvers and provide the basic interfaces for solving differential equations in theoretical models.

### 4.2. Domain Programming Interface

In this section, we specify the domain programming interfaces that are closely related to the solver implementation. It can be concluded that the core of the numerical solution is to discretize the differential equation according to the approaches described in Section 3. After the partial differential equation is discretized using the finite volume method, it can be transformed into a linear equation.

(23) Ax=b

where A is a coefficient matrix and constant vector b is usually called source vector. Column vector x consists of corresponding values on different spatial grids. The theoretical model consists of a series of partial differential equations, each of which includes multiple terms, such as time derivatives, convection terms, Laplace terms, and so on. These items can be separately discretized by the interface functions provided by the framework.

The finite volume method first integrates each term on mesh volumes, which are then linearized in a certain discrete format as part of the linear Equation (23). The discrete format can be specified either by writing a program or by reading the input file at run time. Discrete programming equations in OpenFOAM are defined as static member functions for both classes, finite Volume Method (fvm) and finite Volume Calculus (fvc). The member function of fvm implicitly discretizes the difference equations into the form of a coefficient matrix, which is then added to the left side of the linear system. The member function of fvc returns the corresponding field vector through explicit discretization, eventually joining the right source term.

Major discrete interfaces provided in OpenFOAM are shown in Table 1. The details can be found in the OpenFOAM manual [33].

The function name column contains description of the function parameters, where phi can represent various types of body fields, including scalar, vector, and tensor types; rho is a scalar field; psi is a surface field that can be derived from interpolating the value stored in the volume center into the surface through the finite volume method; chi can represent a surface field or a body field. Note that highest dimension of the data type is a 9-dimensional tensor field, so the gradient term can not operate on the tensor field.

The differential operators corresponding to the governing equations in the solver are included in Table 1, so we can implement the discretization of theoretical models based on the above programming interface.

### 4.3. Implementation

**Figure 2:**
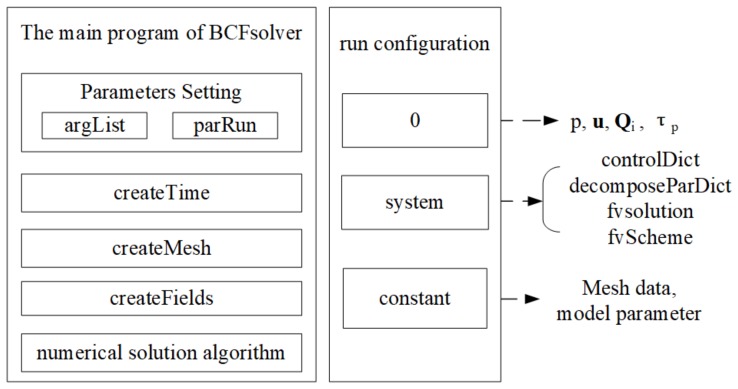
Numerical solver module diagram.

The numerical solver, BCFsolver, consists of two parts. The core part is the solver’s main program, as displayed in the left side of Figure 2. Before starting the iteration of the numerical solution algorithm, the main program first needs to read the input parameters through the “argList” module. If the current program is executed in parallel, “parRun” would be called to start the parallel process. “parRun” calls initialization interface of the underlying parallel library to start MPI and complete the initialization of the process and the communication domain. “createTime” initializes the simulation program’s time control module, which controls the total simulation time, the time step size and so on. The “createMesh” module constructs the grid data structure based on the grid information entered. All the physical variables in the program are constructed based on the grid data structure. Therefore, the “createMesh” module must be initialized before “createFields”. “createFields” initializes the various field variables needed in the simulation based on the grid data, including pressure field *p*, velocity field u, viscoelastic stress field $\tau_{p}$. The numerical solution algorithm module implements the iterative solution process described in Algorithm 1.

The main program of the BCFsolver also needs dynamic running configuration information for execution. These running configurations include three parts, which are respectively stored in the “0”, “system” and “constant” file directories. The “0” directory defines the value of each field variable at the initial time. The “system” directory defines the key configuration information for solving the system, which includes the four parts shown in Figure 2. The “constant” folder is configured with mesh data and model parameters.

The controlDict file contains process control variables of the simulation program. The start time of the simulation is generally set to 0. You can also continue from the last simulation saved interrupt. The end time determines the total physical length of the simulation process. The deltaT determines the time step of discrete simulation. To ensure the accuracy of simulation, the time step can not be set too large. It is generally considered that the largest time step should ensure that the fluid advancement distance should not be greater than the width of one grid in a single time step. Therefore, Courant number is defined as $Co=\left.v_{m}\Delta t/\Delta l\right.$ to describe the ratio of the distance of fluid movement to the width of the grid in a time step, where $v_{m}$ is the maximum fluid velocity, Δt is the size of the time step, and Δl is the mesh width. The smaller the Courant number, the higher the time accuracy of the simulation, leading to heavy computing burden. The maximum Courant number is significantly less than one in general. The Courant number is an important reference for setting the time step. Therefore, the controlDict provides the option of dynamically adjusting the time step according to the value of Co. We can set a maximum Courant number “maxCo” and a maximum time step “maxDeltaT”, and the size of the time step is dynamically adjusted depending on the fluid velocity. The simulation results need to be written to a file for post-processing. The writeInterval parameter defines the interval at which data is written.

The decomposeParDict file defines a grid-based parallel division. Different partitioning methods can be used to decompose the grid data into individual processors, depending on the complexity of the geometry of the simulation problem.

The fvSolution and fvScheme respectively define the linear equations solver used in the algorithm and numerical discrete format. The most basic linear equations solving algorithm is the conjugate gradient method, and the numerical solver implemented in this paper mainly uses the algorithm. The specific configuration is shown in Table 2.

The essence of a FVM calculation process is to satisfy the conservation law by integrating each differential term over the control volume in the equation. The numerical discretization of equation items determines the process of transforming field data on discrete grids into a system of linear equations, which has an important influence on the accuracy and numerical stability of the solution process. Discrete formats used in the solver are displayed in Table 3.

Constant directory contains two parts of information. The first part is the definition of the model constants. All parameters can be set in the transportProperties, such as relaxation time, viscosity, fluid density and so on. The second part is the grid data definition.

## 5. Results and Discussion

We ran multi-scale simulations with different configurations on a cluster located in the State Key Laboratory of High Performance Computing of National University of Defense Technology. In this cluster, each computing node contains 12 Intel Xeon E5-2620 2.10 GHz CPU cores and a total main memory of 16 GB. Each calculation presented in the paper costs around 2∼7 days on 8 CPU cores.

### 5.1. Problem Specification

**Figure 3:**
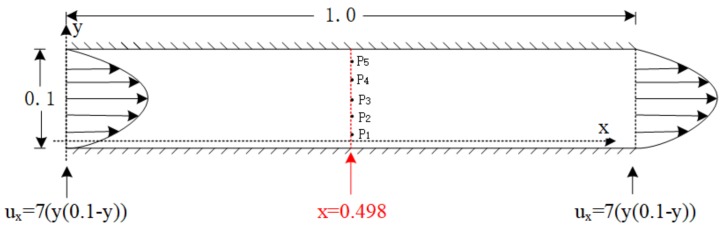
Poiseuille flow in a planar channel and the velocity component $u_{y}=u_{z}=0$. We perform measurements on the line x=0.498 or at the grid points $P_{1}=\left(0.498,0.01\right)$, $P_{2}=\left(0.498,0.03\right)$, $P_{3}=\left(0.498,0.05\right)$, $P_{4}=\left(0.498,0.07\right)$ and $P_{5}=\left(0.498,0.09\right)$.

For an unsteady Poiseuille flow in a planar channel, we assume no-slip boundary conditions at the channel wall. We employ a channel of length 1.0 in the *x*-direction, height 0.1 in the *y*-direction and of width 0.01 in the *z*-direction. We prescribe the velocity component $u_{x}$ at the inlet and outlet boundary. Figure 3 shows the two-dimensional geometry and the initial boundary conditions.

The parameters of the micro-macro model need to be set in the configuration file, “transportProperties”, located in the “constant” folder. Some of the parameters remain constant throughout all of the simulations and are displayed in Table 4:

In the simulations, we test the designed micro-macro numerical solver on different meshes. Table 5 lists the different number of grid cells and the corresponding mesh width used in our simulations.

### 5.2. Simulation Results

Since the stochastic approach is much more demanding in terms of memory and computing resources, we first perform the simulations on the mesh level l=2. The fluid density ρ=0.1 at first. And we compare the results for the Oldroyd-B model, the Hooke model and the FENE model. For the Hookean dumbbell model and FENE model, the number of stochastic realizations $N_{\mathrm{BCF}}=800$ and the dumbbell’s extension parameter b=100. Then we test the Hookean dumbbell model for different parameters or different mesh levels. At last, we concentrate on the FENE model and investigate this model under different conditions. More importantly, we give the molecular distribution and stretch of 2D viscoelastic Poiseuille flow.

**Figure 4:**
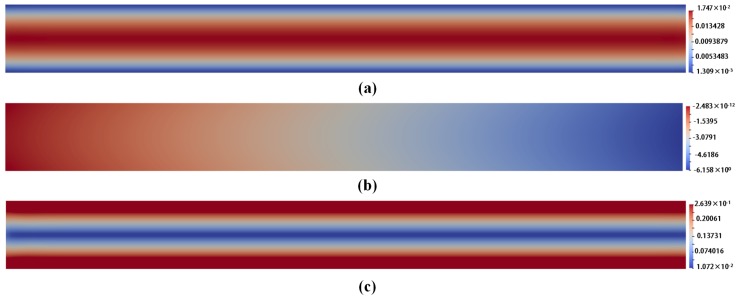
The snapshots: (**a**) of the velocity field u for Oldroyd-B model with l=2,ρ=0.1 at t=1.999185; (**b**) of the pressure field *p* for Oldroyd-B model with l=2,ρ=0.1 at t=1.999185; and (**c**) of the stress tensor field $\tau_{p}$ for Oldroyd-B model with l=2,ρ=0.1 at t=1.999185.

**Figure 5:**
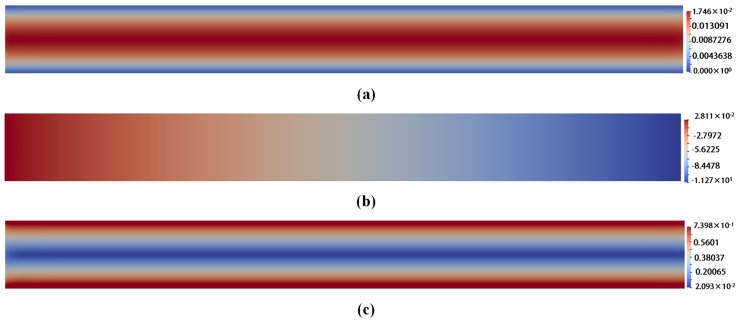
The snapshots: (**a**) of the velocity field u for Hooke model with $l=2,\rho =0.1,N_{\mathrm{BCF}}=800$ at t=1.999185; (**b**) of the pressure field *p* for Hooke model with with $l=2,\rho =0.1,N_{\mathrm{BCF}}=800$ at t=1.999185; and (**c**) of the stress tensor field $\tau_{p}$ for Hooke model with with $l=2,\rho =0.1,N_{\mathrm{BCF}}=800$ at t=1.999185.

**Figure 6:**
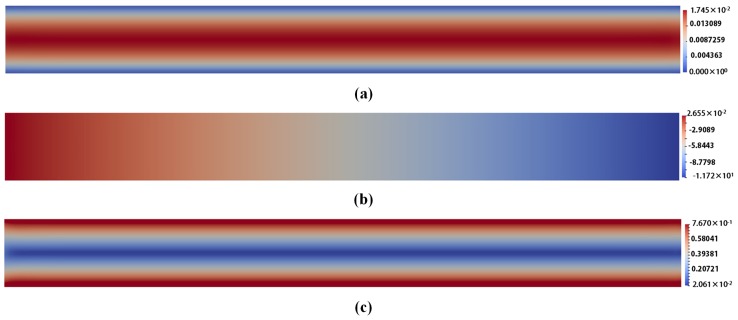
The snapshots: (**a**) of the velocity field u for FENE model with $l=2,\rho =0.1,N_{\mathrm{BCF}}=800$, b=100 at t=1.999185; (**b**) of the pressure field *p* for FENE model with $l=2,\rho =0.1,N_{\mathrm{BCF}}=800$, b=100 at t=1.999185; and (**c**) of the stress tensor field $\tau_{p}$ for FENE model with l=2,ρ=0.1, $N_{\mathrm{BCF}}=800,b=100$ at t=1.999185.

The snapshots of different field variables for corresponding models are illustrated from Figure 4, Figure 5 and Figure 6. For comparison, the visualized time points in these figures are chosen in a steady state at t=1.999185.

From Figure 4, Figure 5 and Figure 6, we can draw a conclusion that the snapshots of u and *p* fields are almost the same for the three models. However, for the stress tensor field $\tau_{p}$, the snapshot of $\tau_{p}$ for Oldroyd-B model is different from that for the other two models. For the Hookean dumbbell and FENE model, it is obvious that the $\tau_{p}$ is not so symmetric as that for the Oldroyd-B model. The Brownian configuration field method introduces stochastic noise and the $\tau_{p}$ is derived from the average of local ensemble of sample particles through mathematical statistics. It might lead to additional error to the solution. As a result, the noise-free macroscopic model yields better results than the multi-scale approach.

**Figure 7:**
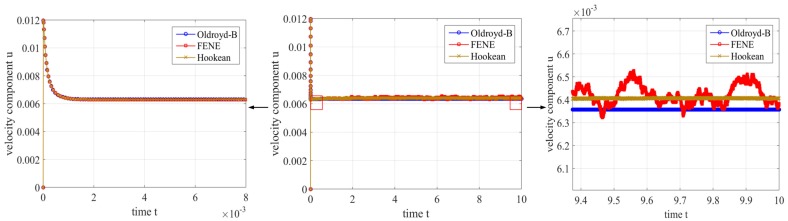
The evolution of horizontal velocity component $u_{x}$ for different models (Oldroyd-B model, Hooke model and FENE model) over time at point $P_{1}=\left(0.498,0.01\right)$.

In Figure 7, we examine the velocity field u at position $P_{1}=\left(0.498,0.01\right)$ over time. The curve of velocity component $u_{x}$ is plotted with zoomed pictures. And the zoomed region is indicated with red rectangles in the full picture. Compared to the macroscopic result, the curves of multi-scale methods for Hooke and FENE model show minor changes caused by stochastic noise. But they all exhibit overshoot at the beginning of the simulation. We cannot expect to yield better results for the multi-scale model than for the corresponding macroscopic model because of the introduced stochastic noise. However, it is possible that the simulation with higher $N_{\mathrm{BCF}}$ performs better with respect to the macroscopic Oldroyd-B solution.

#### 5.2.1. Hooke Model

**Figure 8:**
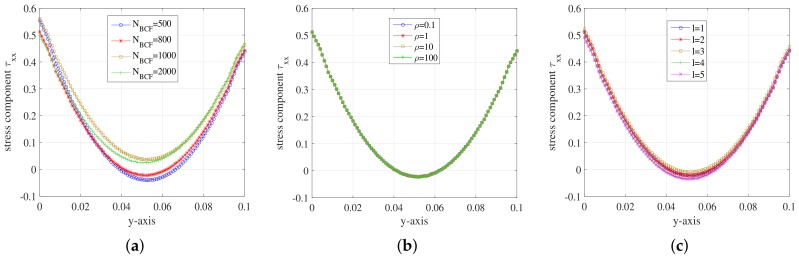
Stress component $\tau_{\mathrm{xx}}$ on the vertical channel wall during the steady state at t=1.999185: (**a**) for different $N_{\mathrm{BCF}}$ of Hooke model; (**b**) for different Re number of Hooke model; and (**c**) for different mesh levels of Hooke model.

Since all unknowns solely depend on y when the unknown fields evolve into steady state, we display the unknowns in a steady state of the macroscopic and Hookean dumbbell models along the *y*-axis. To investigate the effects of different situations, we change only one parameter every time we test the effect of corresponding one. For example, we change the number of stochastic realizations $N_{\mathrm{BCF}}$ and the other parameters remain the same with those set in Figure 5. Analogously, we adjust *R*e (Reybolds number) through changing the density of the fluid. We also change the mesh levels according to Table 5 and carry on the simulation for the Hookean dumbbell model on different mesh levels. Figure 8 shows the evolution of stress component $\tau_{\mathrm{xx}}$ along *y*-axis for different parameters of the Hookean dumbbell model.

In Figure 8a, the minima of these curves increase as the number of stochastic realizations $N_{\mathrm{BCF}}$ becomes larger. The minima of curve at $N_{\mathrm{BCF}}=2000$ is close to 0 and the symmetry improved a lot. It can be concluded that the errors of simulation results become smaller with the increase of $N_{\mathrm{BCF}}$. Figure 8b shows that the curves of stress tensor component $\tau_{\mathrm{xx}}$ have no difference among each other. We can draw a conclusion that the value of Re number imposes little effect on the simulation of Hooke model. In Figure 8c, these curves are close to each other for different mesh levels although there are some errors. The results of stress component $\tau_{\mathrm{xx}}$ for Hooke model show systematic deviations compared to the equivalent Oldroyd-B constitutive model. Hooke model introduces stochastic noises. However, we employ low-order temporal discretization schemes which may impose less control on the noises than the higher ones. We will explore the reason in further study.

**Figure 9:**
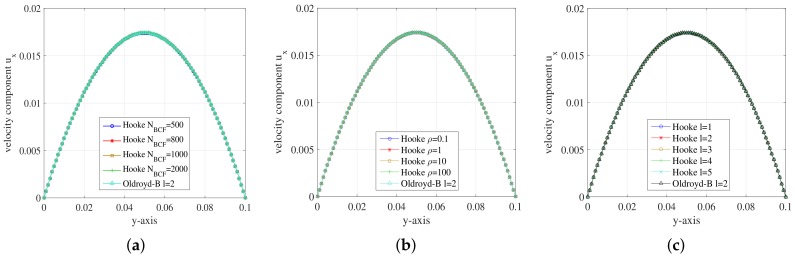
Velocity component $u_{x}$ on the vertical channel wall during the steady state at t=1.999185: (**a**) for different $N_{\mathrm{BCF}}$ of Hooke model and Oldroyd-B model l=2; (**b**) for different Re number of Hooke model and Oldroyd-B model l=2; and (**c**) for different mesh levels of Hooke model and Oldroyd-B model l=2.

Figure 9 shows the evolution of velocity component $u_{x}$ along *y*-axis for different parameters of the Hookean dumbbell model. For comparison, we also present the simulation result of the equivalent Oldroyd-B constitutive model in corresponding graphs.

From Figure 9, we can see that the velocity component $u_{x}$ of Hooke model achieved good consistency with the Oldroyd-B model. It can be concluded that the Hooke model can be employed in the simulation for the flow behaviour in 2D planar channel.

#### 5.2.2. FENE Model

**Figure 10:**
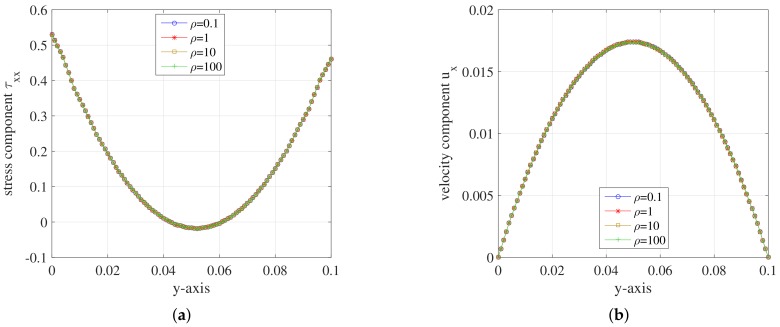
(**a**) Stress component $\tau_{\mathrm{xx}}$; and (**b**) velocity component $u_{x}$ on the *y*-axis during the steady state at t=1.999185 for different Re number of FENE model.

For the FENE model, we get the simulation results about stress tensor component $\tau_{\mathrm{xx}}$ and velocity component $u_{x}$ for different conditions along the *y*-axis. We first change the Re number of the FENE model by adjusting the density of the polymer solutions. At the meantime, the stochastic realizations are set $N_{\mathrm{BCF}}=800$ per grid cell and the simulation is implemented on the mesh level l=2. For the FENE model, the dumbbell’s extension parameter *b* is restricted and we set the extension parameter b=100. The results are presented in Figure 10.

In Figure 10, the curves of stress tensor component $\tau_{\mathrm{xx}}$ have no difference among each other and so does velocity component $u_{x}$. It can be concluded that the value of Re number imposes little effect on the simulation of the FENE fluid flow in a planar channel.

**Figure 11:**
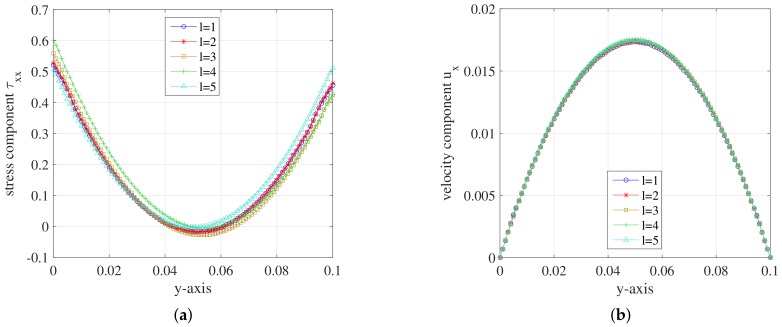
(**a**) Stress component $\tau_{\mathrm{xx}}$; and (**b**) velocity component $u_{x}$ on the *y*-axis during the steady state at t=1.999185 for different mesh levels of FENE model.

We change the mesh levels according to Table 5. We set $\rho =100,N_{\mathrm{BCF}}=800,b=100$ again for the simulation. The simulation results of stress tensor component $\tau_{\mathrm{xx}}$ and velocity component $u_{x}$ on different mesh levels are presented and compared in Figure 11.

For the stress tensor component $\tau_{\mathrm{xx}}$, the symmetry of the curve becomes better as the number of mesh cells increases. For the mesh level l=5, the curve of $\tau_{\mathrm{xx}}$ component performs the best among these mesh levels. The curves of velocity component $u_{x}$ are close to each other for different mesh levels. The $u_{x}$ component at y=0.5 becomes closer to the maximum u=0.0175 as the cell number increases. It can be concluded that the simulation results perform better with the increase of mesh density.

**Figure 12:**
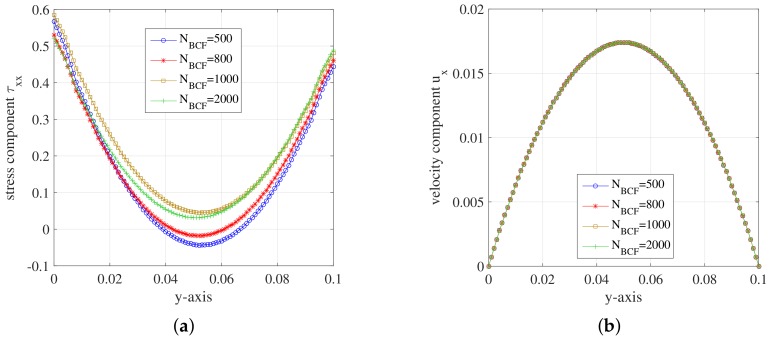
(**a**) Stress component $\tau_{\mathrm{xx}}$; and (**b**) velocity component $u_{x}$ on the *y*-axis during the steady state at t=1.999185 for different $N_{\mathrm{BCF}}$ of FENE model.

To investigate the effect of the number of stochastic realizations $N_{\mathrm{BCF}}$, we set the parameters ρ=0.1,b=100 and the simulations run on mesh level l=2. Figure 12 displays the stress tensor component $\tau_{\mathrm{xx}}$ and velocity component $u_{x}$ along the *y*-axis.

For the stress tensor component $\tau_{\mathrm{xx}}$, the minima of these curves increase as the value of $N_{\mathrm{BCF}}$ becomes larger. Compared with $N_{\mathrm{BCF}}=1000$, the symmetry of the curve at $N_{\mathrm{BCF}}=2000$ has improved. The curves of $u_{x}$ component coincide with each other. From Figure 12, we can draw a conclusion that the accuracy of the solutions to the FENE model improved as $N_{\mathrm{BCF}}$ increases.

**Figure 13:**
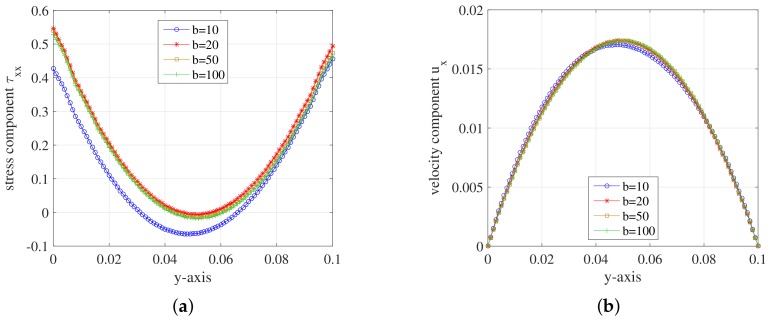
(**a**) Stress component $\tau_{\mathrm{xx}}$; and (**b**) velocity component $u_{x}$ on the *y*-axis during the steady state at t=1.999185 for different dumbbell’s extension parameter *b* of FENE model.

We also investigate the effect of the dumbbell’s extension parameter *b*. The other parameters are set as follows: $\rho =0.1,N_{\mathrm{BCF}}=800$. Again, the simulations run on the mesh level l=2. Figure 13 displays the results.

The curves of the stress tensor component $\tau_{\mathrm{xx}}$ nearly coincide with each other when b>10. Compared with other *b* values, the values of $\tau_{\mathrm{xx}}$ component are bigger than that of b=10. It means that the molecules are extended longer with larger dumbbell’s extension parameter. Corresponding to the result of the $\tau_{\mathrm{xx}}$ component, the curve of $u_{x}$ component of b=10 deviates from the curves of other values of *b*. In order to study the stretch of molecules in FENE model, we set the dumbbell’s extension parameter b=100 in the simulations of Section 5.3.

The results of the FENE model with the dumbbell’s extension parameter b=100 is very close to the Hooke model for the present conditions. So we test and make a comparison. The simulations run on the mesh level l=2 with ρ=0.1 and $N_{\mathrm{BCF}}=800$ for both models. The parameters remaining constant throughout the simulations are displayed in Table 4.

**Figure 14:**
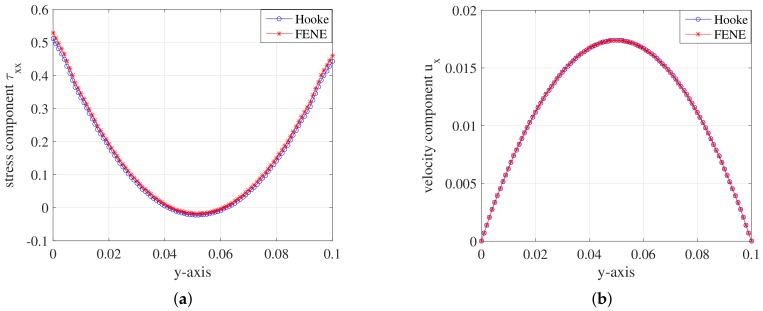
(**a**) Stress component $\tau_{\mathrm{xx}}$; and (**b**) velocity component $u_{x}$ on the *y*-axis during the steady state at t=1.999185 for FENE model with the dumbbell’s extension parameter b=100 and Hooke model.

As Figure 14 shows, both the stress component $\tau_{\mathrm{xx}}$ and the velocity component $u_{x}$ are very close to each other. It can be concluded that the FENE model with the dumbbell’s extension b=100 displays almost the same as the Hooke model in the simulation of the flow behaviour in 2D planar channel.

### 5.3. Molecular Distribution

To investigate the molecular distribution and stretch of 2D viscoelastic Poiseuille flow, We designed a new scalar Field *D* to describe the length of the extended molecules. We called the scalar Field *D* as “stretch length”. The stretch length *D* is defined as:

(24) $$D=∥Q_{t}∥=\sqrt{Q_{t(x)}^{2}+Q_{t(y)}^{2}+Q_{t(z)}^{2}}$$

where $Q_{t(x)}$, $Q_{t(y)}$, $Q_{t(z)}$ represent the component of $Q_{t}$ in the *x*, *y*, *z* directions, respectively.

**Figure 15:**
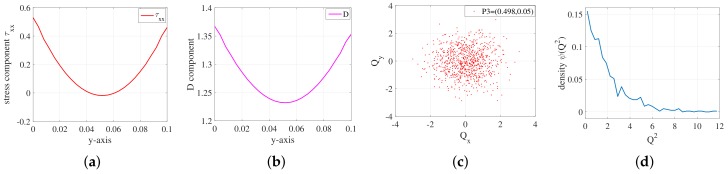

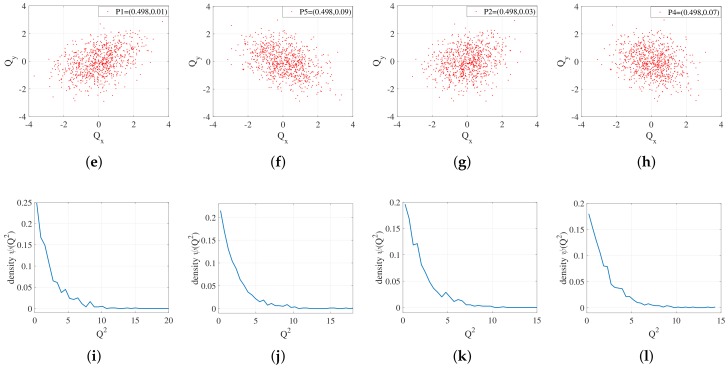
(**a**) Stress component $\tau_{\mathrm{xx}}$ on the *y*-axis; (**b**) stretch length *D* on the *y*-axis; (**c**) the distribution of molecules $Q_{3}$ at point $P_{3}=\left(0.498,0.05\right)$; (**d**) corresponding probability density function (pdf) at point $P_{3}=\left(0.498,0.05\right)$; (**e**) the distribution of molecules $Q_{1}$ at point $P_{1}=\left(0.498,0.01\right)$; (**f**) the distribution of molecules $Q_{5}$ at point $P_{5}=\left(0.498,0.09\right)$; (**g**) the distribution of molecules $Q_{2}$ at point $P_{2}=\left(0.498,0.03\right)$; (**h**) the distribution of molecules $Q_{4}$ at point $P_{4}=\left(0.498,0.07\right)$; (**i**) corresponding probability density function (pdf) at point $P_{1}=\left(0.498,0.01\right)$; (**j**) corresponding probability density function (pdf) at point $P_{5}=\left(0.498,0.09\right)$; (**k**) corresponding probability density function (pdf) at point $P_{2}=\left(0.498,0.03\right)$; and (**l**) corresponding probability density function (pdf) at point $P_{4}=\left(0.498,0.07\right)$.

We implement the simulation with the parameters $\rho =0.1,N_{\mathrm{BCF}}=800,b=100$ on mesh level l=2 for FENE model. The stress tensor component $\tau_{\mathrm{xx}}$ and corresponding *D* component along the *y*-axis, as well as the distribution of the molecules and corresponding probability density function (pdf), are presented in Figure 15.

$P_{3}$ is located in the center of the planar channel; $P_{1},P_{2}$ and $P_{4},P_{5}$ are vertically symmetrically located on both sides of $P_{3}$. The curve of $\tau_{\mathrm{xx}}$ component shows that the value of $\tau_{\mathrm{xx}}$ at $P_{3}$ is very close to 0. It means that the molecular distribution there was nearly not stretched out. As a result, the distribution of the molecules at $P_{3}$ remain in line with the normal distribution. However, the values of $\tau_{\mathrm{xx}}$ component at $P_{1}$, and $P_{5}$ are nearly the same and both are bigger than 0. Therefore, the molecular distribution there are stretched and the molecules are distributed symmetrically. And so do $P_{2}$ and $P_{4}$.

## 6. Conclusions

The BCF method provides a new perspective to study the rheological characteristics of polymer solutions using a multi-scale numerical solver. In this paper, we gave a full multi-scale model, which includes the macroscopic equations governing the hydrodynamics of the Newtonian solvent, and the microscopic equations governing the motion of the polymer molecules in the solution.

To solve the governing equations numerically, a multi-scale solver was implemented based on the open source CFD (Computional Fluid Dynamics) toolbox, OpenFOAM. The architecture of the solver and the numerical techniques used in the solver were discussed in detail. The simple two-dimensional Poiseuille flow with various configurations was run as the baseline. The key variable in the simulation, such as the velocity component $u_{x}$, achieved good consistency with macroscopic counterpart. What’s more, we designed a new scalar Field *D* to quantitatively describe the local stretch of molecules, thus the global correlations between the molecular distributions and the macroscopic polymer solutions could be analyzed.

The multi-scale simulation results of the OpenFOAM-based solver showed great potential to study complex engineering problems involving dilute polymer solutions. In the future, we will apply this approach to three-dimensional complex geometries. In order to reduce the computation time, we will transport the multi-scale solver to high performance clusters and focus on parallel optimization techniques.

## Figures and Tables

**Table 1:** The main partial differential equations discrete programming interface in OpenFOAM.

Differential term	Explicit/implicit	Model expressions	Function name
laplace term	explicit/implicit	$\nabla^{2}\phi$	laplacian(phi)
		∇·Γ∇ϕ	laplacian(Gamma,phi)
time derivative term	explicit/implicit	$\frac{\partial \phi }{\partial t}$	ddt(phi)
		$\frac{\partial \rho \phi }{\partial t}$	ddt(rho,phi)
2-nd order time derivative term	explicit/implicit	$\frac{\partial }{\partial t}\left(\rho \frac{\partial \phi }{\partial t}\right)$	d2dt2(rho,phi)
convection term	explicit/implicit	∇·(ψ)	div(psi,scheme)
		∇·(ψϕ)	div(psi,phi,word)
			div(psi,phi)
divergence term	explicit	∇·χ	div(chi)
gradient term	explicit	∇χ	grad(chi)
		∇ϕ	gGrad(phi)
			lsGrad(phi)
			snGrad(phi)
			snGradCorrection(phi)
source term	implicit	ρϕ	Sp(rho,phi)

**Table 2:** The basic configuration of linear equations solver.

Field variables	Solver	Preprocessor	Error limit
p	PCG	GAMG	$10^{-8}$
u	PBiCG	GAMG	$10^{-8}$
$\tau_{p}$	BICCG	DILU	$10^{-7}$
$Q_{i}$	PBiCG	DILU	$10^{-10}$

**Table 3:** Numerical discrete format used in the solver.

Equation terms	Discrete format	Accuracy
First-order time derivative	Euler	1st-order
Gradient	Gauss linear	2nd-order
Divergence	Gauss Minmod/linear	2nd-order
Laplace	Gauss linear corrected	2nd-order

**Table 4:** Parameters used in simulations.

Parameter	$\eta_{s}$	$\eta_{p}$	$W_{i}$	λ
Value	0.05	0.40	1.0	0.6

**Table 5:** Mesh characteristics on different levels l used for the simulations.

l	$\Delta x_{l}$,$\Delta y_{l}$	$\Delta z_{l}$	Cells/direction	Total cell
1	$5\times 10^{-3}$	0.01	200×20×1	4000
2	$4\times 10^{-3}$	0.01	250×25×1	6250
3	$2.5\times 10^{-3}$	0.01	400×40×1	16,000
4	$2\times 10^{-3}$	0.01	500×50×1	25,000
5	$1.25\times 10^{-3}$	0.01	800×80×1	64,000
